# Cancer is associated with inferior outcome in patients with ischemic stroke

**DOI:** 10.1007/s00415-021-10528-3

**Published:** 2021-05-04

**Authors:** Katharina Seystahl, Alessia Hug, Sung Ju Weber, Sandra Kapitza, Dorothee Gramatzki, Miriam Wanner, Mira Katan, Andreas R. Luft, Sabine Rohrmann, Susanne Wegener, Michael Weller

**Affiliations:** 1 Department of Neurology, University Hospital and University of Zurich, CH-8091 Zurich, Switzerland; 2 Cancer Registry of the Canton of Zurich, Zug, Schaffhausen and Schwyz, University Hospital and University of Zurich, Zurich, Switzerland; 3 Cereneo Center for Neurology and Rehabilitation, Vitznau, Switzerland

**Keywords:** Stroke, Risk factors, Cancer, Sex

## Abstract

**Background:**

Whether patients with stroke and cancer exhibit specific characteristics has remained controversial.

**Methods:**

Medical records of patients with ischemic stroke in 2014 or 2015 registered in the Swiss Stroke Registry of Zurich were retrospectively analyzed and integrated with regional cancer registry data. Associations of clinical and outcome parameters with cancer diagnosed up to 5 years prior to stroke were tested.

**Results:**

Of 753 patients with ischemic stroke, 59 patients with cancer were identified. History of venous thromboembolism (*p* < 0.001) was associated with cancer while age and cardiovascular risk factors were not. Higher levels of D-dimers (*p* = 0.001), erythrocyte sedimentation rate (*p* = 0.003), C-reactive protein (CRP) (*p* < 0.001), and lower levels of hemoglobin (*p* = 0.003) were associated with cancer. For platelets, pathologically low (*p* = 0.034) or high levels (*p* < 0.001) were linked to cancer. Modified Rankin scale (mRS) scores ≥ 4 on admission and at follow-up were more frequent in cancer patients (*p* = 0.038 and *p* = 0.001). Poor post-stroke survival was associated with cancer (HR 2.2, *p* < 0.001). Multivariable analysis identified venous thromboembolism (OR 5.1), pathologic platelet count (OR = 2.9), low hemoglobin (OR 2.5) and elevated CRP (OR 1.8) as independently associated with cancer. In multivariable Cox regression, risk for death was associated with cancer (HR 1.7), low hemoglobin (HR 2.6), mRS on admission ≥ 4 (HR 1.9), pathologic platelet count (HR 1.6), female sex (HR 1.7), and elevated CRP (HR 1.4).

**Conclusions:**

Considering cancer as a cofactor for post-stroke outcome may impact clinical decision making.

**Supplementary Information:**

The online version contains supplementary material available at 10.1007/s00415-021-10528-3.

## Introduction

Cancer and stroke represent frequent causes for morbidity and mortality in Western countries [1–3]. An epidemiologic study reported an elevated risk for stroke particularly within 6 months, but up to 10 years after cancer diagnosis [4]. Autopsy of 3426 cancer patients revealed cerebrovascular disease in 500 (14.6%) of patients. In 245 (49%) of them, strokes were clinically silent [5]. Various studies evaluated stroke etiology, risk profiles and clinical characteristics of patients with ischemic stroke and cancer [6–11]. History of venous thromboembolism was associated with cancer in several studies of stroke patients, suggesting a pro-thrombotic state in cancer patients [7, 9, 12]. Several laboratory parameters were linked with cancer in stroke patients, including elevated D-dimer levels [7, 10, 13], low hemoglobin [10], higher levels of C-reactive protein (CRP), and higher erythrocyte sedimentation rate (ESR) [14]. Regarding imaging, incidence of ischemic lesions in multiple vessel territories were related to cancer in stroke patients [7]. However, whether large vessel occlusions are more prevalent in stroke patients with cancer remains uncertain. Stroke patients with cancer had higher in-hospital mortality [15–18], however, the availability of longitudinal data is limited.

The current study provides a comprehensive analysis regarding patient and family history, clinical characteristics and outcome, laboratory parameters, imaging findings, also taking into account sex-specific characteristics of patients with ischemic stroke without or with known cancer up to 5 years prior to stroke.

## Patients and methods

### Patients and variables

**Fig. 1:**
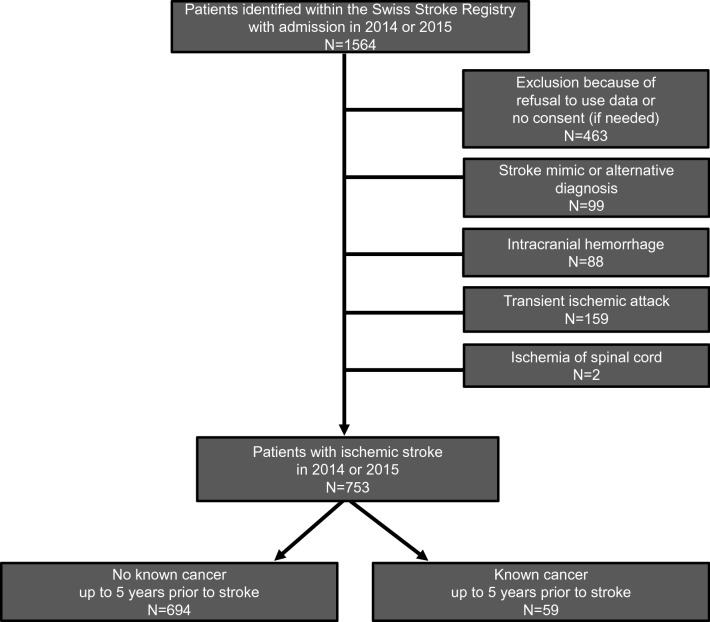
Consort chart. Shown is the selection process for identification of the study population

Patients admitted for stroke or transient ischemic attack (TIA) to the University Hospital Zurich in 2014 and 2015 were identified within the Zurich Swiss Stroke Registry patient cohort. For this study, patients with ischemic stroke as defined by the American Stroke Association as brain infarction attributable to ischemia and based on neuroimaging, and/or clinical evidence of permanent injury [19] were analyzed with consent available or not needed according to the requirements of the institutional review board Cantonal ethics committee of Zurich approval (KEK-ZH 2018–01,917). Figure 1 shows the process of subject identification. The documentation of the Swiss Stroke Registry was supplemented by retrospective patients’ chart review. In patients with recurrent admissions for stroke within the analyzed period, only the first event was used. “Known cancer” was recorded if any neoplastic disease was documented in the clinical chart excluding benign tumors such as adenomas as well as basal cell carcinoma, schwannomas and meningiomas because of the presumable lack of systemic cancer effects. Data on cancer incidence and follow-up were derived from the medical records for all patients and additionally matched with the data of the Cancer Registry of the Cantons Zurich, Zug, Schaffhausen and Schwyz in Switzerland for 585 of 753 patients.

To group patients, we chose as cut-off any cancer diagnosis up to 5 years prior to stroke based on a previously reported increased risk of stroke up to 10 years after cancer diagnosis [4], and also included the period of in-hospital work-up for stroke.

Active or previous smoking was documented if any smoking was noted in the clinical chart and quantified by pack years if available. Stroke etiology was assessed by the TOAST classification [20] based on the documentation in the clinical chart. Data on stroke severity via National Institutes of Health Stroke Scale (NIHSS) on admission and at approximately 24 h after admission as documented in the clinical chart was used as well as degree of disability via modified Rankin scale (mRS) prior to stroke, on admission and at follow-up. mRS data were derived from the medical records or the Swiss stroke registry or both if suitable information was available either from reports of a clinical visit or of phone calls to the patients or their relatives or from available documents including reports of other hospitals or notes of deaths where applicable. Since no preplanned follow-up visit was available due to the retrospective study design, only follow-up data on mRS documented between 60 and 120 days after stroke were used, also including patients that died at any day up to 120 days after stroke. Post-stroke survival was calculated from the date of stroke to death or to the date of last contact as available in the clinical chart. Patients were censored at last follow-up if survival status was unknown. Cause of death was categorized based on the information available in the medical reports into (1) cardiovascular etiology including complications of stroke, (2) cancer, (3) other or (4) unknown. Regarding imaging characteristics, ischemic lesions were categorized to be present in < 2 or ≥ 2 vessel territories and incidence of large vessel occlusions was analyzed by review of the radiological report and/or review of images by central nervous system (CNS) magnetic resonance imaging (MRI) or computed tomography (CT) as applicable.

Regarding laboratory parameters, the first value available after admission was included in the analysis. Levels of hemoglobin, D-dimers, lactate dehydrogenase (LDH), ESR, CRP, total cholesterol, low density lipoprotein (LDL), thyroid stimulatory hormone, creatinine and glucose were used as continuous linearly scaled parameters for univariable analyses. Values for CRP and ESR were only included if the analysis was done within 24 h after admission. Values of D-dimers were excluded if blood was drawn after intravenous thrombolysis. White blood count (WBC) and platelet count were analyzed as categorical parameters using the local reference standards of upper level of normal (ULN) for WBC grouping them into ≤ 9600/μl or > 9600/μl and lower level of normal (LLN) and ULN for platelet count, i.e. grouping values into < 143,000/μl, ≥ 143,000/μl and ≤ 400,000/μl or > 400,000/μl. For binary multivariable analyses the following local reference standards were used as cut-offs, i.e. LLN of hemoglobin 117 g/l for women and 134 g/l for men, respectively, and 5 mg/dl as ULN for CRP. The choice of LLN and/or ULN as cut-offs was arbitrarily and did not represent a pre-specified analysis.

### Statistical analysis

Patients’ characteristics were analysed by descriptive statistics. For univariable analyses, Chi-square test was applied for categorical variables and Mann–Whitney *U* tests for ordinal and linear variables, respectively, with a *p* value of ≤ 0.05 defined as statistically significant. Since this was an explorative signal-seeking study, no correction for multiple comparisons was applied. Survival curves were calculated by the Kaplan–Meier method and compared by log-rank test. Binary multivariable logistic regression analysis was performed to test the association of candidate parameters with cancer. Univariable and multivariable Cox proportional hazard regression was done to evaluate the association of parameters with risk of death after ischemic stroke. A term of interaction was included where indicated. Multivariable models were calculated for the subgroup of patients with all tested co-variables available. Statistical analyses were performed using SPSS Statistics, Version 26 and Graphpad Prism, Version 8.0.

## Results

### Patient characteristics

**Fig. 2:**
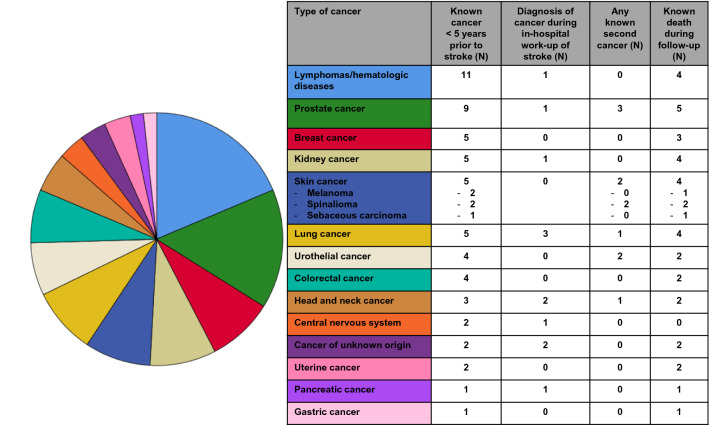
Characteristics of patients with known cancer diagnosed up to 5 years prior to stroke. The pie chart shows proportions of cancer types as specified by the respective colors and data outlined in column 1 of the table. Included were patients diagnosed up to 5 years prior to ischemic stroke as well as during the period of hospitalization for stroke. For the 12 patients with cancer diagnosed during hospitalization, the respective tumor types are listed in column 3. Column 4 shows how many patients received any diagnosis of any second cancer at any time point prior to stroke. The last column indicates how many patients of the respective cancer type died during follow-up

Of 753 evaluable patients with ischemic stroke with a median follow-up of surviving patients of 2.5 years, 59 patients had been diagnosed with cancer within the time frame of up to 5 years prior to stroke (Fig. 1). Figure 2 gives an overview on the tumor types, further characteristics including data on metastases and previous tumor-related treatment are summarized in Table 1. The most prevalent tumors were lymphomas including hematologic diseases and prostate cancer. Twelve of the 59 patients with cancer received the diagnosis during the in-hospital work-up for stroke.

**Table 1:** Patient characteristics of patients with cancer and ischemic stroke

	Known cancer diagnosed up to 5 years prior to stroke *n* = 59 patients
Median time from tumor diagnosis to stroke (years)	1.4 (95% CI 0.7–2.8)
Diagnosis of cancer during in-hospital work-up for ischemic stroke: *n* (%)	12 (20%)
Lymph node metastasis: *n* (%)	
- Yes	17 (29%)
- No	29 (49%)
- No data	13 (22%)
Distant metastasis	
- Yes	12 (20%)
- No	37 (63%)
- No data	10 (17%)
Any tumor-related therapeutic intervention including surgery or systemic therapy or radiotherapy	
- Yes	37 (63%)
- No	18 (31%)
- No data	4 (7%)
Systemic therapy	
- Yes	18 (31%)
- No	17 (29%)
- Number of lines of systemic therapy	
Median (Min–max)	1 (1–6)
- No data	24 (41%)
Radiotherapy	
- Yes	6 (10%)
- No	28 (48%)
- No data	25 (43%)
On a tumor-related therapeutic regimen at the time of ischemic stroke	
- Systemic therapy	9 (15%)
- Radiotherapy	0 (0%)

Patient characteristics for the cohorts without and with cancer are summarized in Table 2. Age and history of cardiovascular risk factors including arterial hypertension, diabetes, hyperlipidemia, smoking, atrial fibrillation, heart disease, previous ischemic stroke, TIA or intracranial hemorrhage were not different between groups. The rate of previous venous thromboembolic events was higher in patients with versus without cancer (*p* < 0.001). Family history of cancer was associated with cancer in stroke patients (*p* = 0.024) while no significant differences were found with regard to family history of ischemic stroke, cardiovascular disease or venous thromboembolism. The rate of patients receiving intravenous thrombolysis was not different between both cohorts while the rate of intraarterial therapeutic interventions without previous intravenous thrombolysis was higher in cancer patients (*p* < 0.001). We asked whether the higher rates of thrombectomy without previous intravenous thrombolysis in cancer patients might reflect that physicians were reluctant to administer intravenous thrombolysis to cancer patients. Indeed, out of 8 patients receiving thrombectomy without previous intravenous thrombolysis, for 2 of them, cancer was documented to be considered for decision against intravenous thrombolysis, for another 2 patients, history of bleeding and for 1 patient previous venous thromboembolism with ongoing anticoagulation were noted as contraindications that may be considered as indirectly related to cancer. One out of the 8 cancer patients receiving an intraarterial therapeutic intervention without previous intravenous thrombolysis suffered a fatal intracranial hemorrhage, while no fatal intracranial hemorrhage was documented in 14 of 59 cancer patients who received intravenous thrombolysis.

**Table 2:** Patient characteristics of patients with ischemic stroke

	No known cancer within 5 years prior to stroke: 694 patients	Known cancer within 5 years prior to stroke: 59 patients	*p* value
Sex (*n*, %)			0.92
Male	395 (56.9%)	34 (57.6%)	
Female	299 (43.1%)	25 (42.4%)	
Age			0.16
Mean ± SD	70 (± 15)	73 (± 12)	
Median (Range)	73 (21–100)	74 (27–91)	
Body mass index			0.82
Median (Range)	25.4 (15.1–54.1)	25.7 (16.7–34.6)	
No data (*n*, %)	25 (3.7%)	3 (5.1%)	
History			
Arterial hypertension	468 (67.4%)	44 (74.6%)	0.26
Diabetes mellitus	103 (14.8%)	13 (22.0%)	0.14
Hyperlipidemia	338 (48.7%)	26 (44.1%)	0.49
Active or previous smoking			0.92
Yes	298 (42.9%)	25 (42.4%)	
No	331 (47.7%)	27 (45.8%)	
No data	65 (9.4%)	7 (11.9%)	
Pack years			0.82
Median (Range)	30 (1–120)	30 (5–90)	
No data	514 (74.0%)	44 (74.5%)	
Atrial fibrillation	140 (20.2%)	16 (27.1%)	0.21
Heart disease (not otherwise specified)	207 (29.8%)	23 (39.0%)	0.14
Ischemic stroke	100 (14.4%)	10 (16.9%)	0.60
Intracranial hemorrhage	18 (2.6%)	3 (5.1%)	0.27
Transient ischemic attack	41 (5.9%)	0 (0%)	0.055
Venous thromboembolism	35 (5.0%)	13 (22%)	* < 0.001
Family history			
Ischemic stroke			0.88
Yes	130 (18.7%)	10 (16.9%)	
No	399 (57.5%)	29 (49.2%)	
No data	165 (23.8%)	20 (33.9%)	
Cardiovascular disease (other than stroke or TIA)			0.28
Yes	162 (23.3%)	9 (15.3)	
No	331 (47.7%)	28 (47.5%)	
No data	201(29%)	22 (37.3%)	
Cancer			*0.024
Yes	94 (13.5%)	13 (22.0%)	
No	246 (35.4%)	14 (23.7%)	
No data	354 (51.0%)	32 (54.2%)	
Venous thromboembolism			0.92
Yes	11 (1.6%)	1 (1.7%)	
No	404 (58.2%)	33 (55.9%)	
No data	279 (40.2%)	25 (42.4%)	
Acute therapy for ischemic stroke			0.51
Intravenous thrombolysis (n, %)	231 (33.3%)	14 (23.7%)	
- With additional intraarterial therapeutic intervention	– 60 (8.6%)	– 5 (8.5%)	
- Without additional intraarterial therapeutic intervention	– 171 (24.6%)	– 9 (15.3%)	
Intraarterial therapeutic intervention without intravenous thrombolysis	26 (3.7%)	8 (13.6%)	* < 0.001

**Fig. 3:**
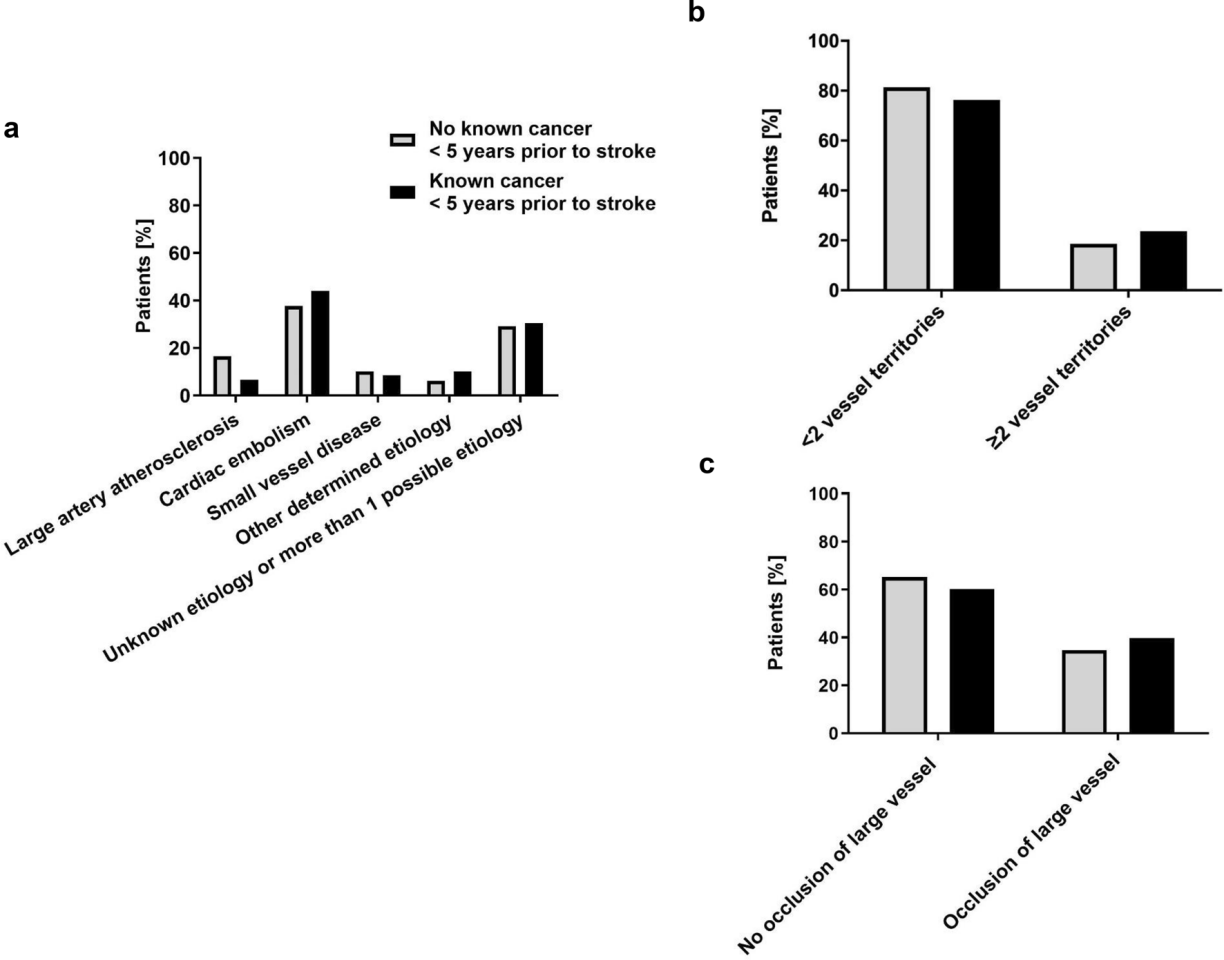
Stroke etiology and imaging parameters of patients with ischemic stroke without and with known cancer. a–c. Shown are stroke etiology as classified by TOAST (**a**), incidence of ischemic lesions in less than 2 or 2 or more vessel territories (**b**) and of large vessel occlusions (**c**) for patients (%) without (grey bars) versus with (black bars) cancer diagnosed up to 5 years prior to stroke

Stroke etiology as classified by TOAST [20] (Fig. 3a) was not different in patients without versus with cancer (*p* = 0.25). Diagnostic workup for stroke etiology scored as cryptogenic stroke was incomplete at the end of the hospitalization for stroke for 37% and 39% of patients without versus with cancer, respectively.

### Imaging characteristics

Ischemic lesions in multiple vessel territories have been related to cancer in stroke patients [7]. In our cohort, we found no significant differences in the incidence of ischemic lesions in < 2 or ≥ 2 or more vessel territories between groups (*p* = 0.33, Fig. 3b). For this analysis, MRI of the CNS at any time point during stroke work-up was available for 83% and 73% of patients without and with cancer while the remaining patients received CT of the CNS. For 6% and 7% of the total cohorts, no ischemic lesion was proven by CT or MRI and stroke diagnosis was based on clinical context.

So far, little is known regarding the incidence of large vessel occlusions in patients with ischemic stroke and cancer, which is particularly interesting in the context of potential tumor-associated paraneoplastic hypercoagulability. However, we found no cancer-associated differences in the incidence of large vessel occlusions in patients with ischemic stroke (Fig. 3c, *p* = 0.45). For this analysis, data were available for 452 (65%) and 43 (73%) patients by CT angiography and for 234 (34%) and 15 (25%) by MR angiography for patients without and with known cancer.

### Laboratory parameters

**Fig. 4:**
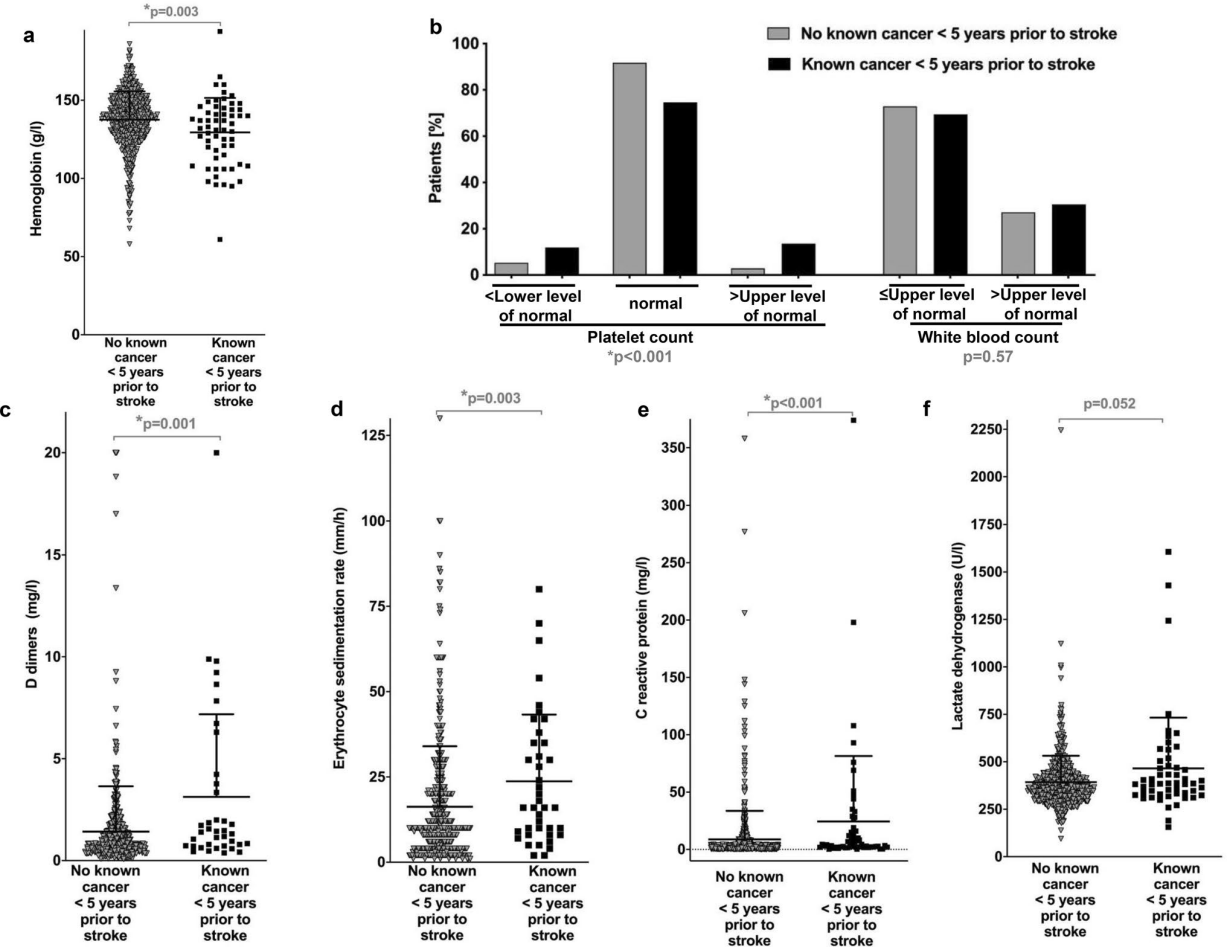
Laboratory parameters of patients with ischemic stroke without and with known cancer. **a**–**f** Laboratory parameters (first measurement available after admission) of 753 patients with ischemic stroke; 694 patients without (grey symbols/bars) and 59 patients with known cancer (black symbols/bars) were analyzed for an association with cancer status. Shown are data including means and SD analyzed by Mann–Whitney *U* test (**a**, **c**–**f**) or percentages of categorial variables analysed by Chi-square test (**b**) for the number of patients without and with cancer who had data on the indicated laboratory parameters available based on retrospective chart review: hemoglobin (**a**, *n* = 694 and *n* = 59 patients), white blood count and platelet count (**b**, *n* = 694 and *n* = 59 patients). D-dimers (**c**, *n* = 487 and *n* = 38 patients), erythrocyte sedimentation rate (**d**, *n* = 463 and *n* = 40 patients), C-reactive protein (**e**, *n* = 690 and *n* = 59 patients), lactate dehydrogenase (**f**, *n* = 574 and *n* = 52 patients)

We further asked whether there were differences in laboratory values between the patient cohorts without versus with cancer (Fig. 4). Lower levels of hemoglobin were associated with cancer (*p* = 0.003, Fig. 4a). Platelet counts categorized into < LLN, ≥ LLN and ≤ ULN or > ULN were different between both cohorts, too (*p* < 0.001) while white blood counts were not (Fig. 4b). Interestingly, both low platelets (< LLN *p* = 0.034) and high platelets (> ULN/μl, *p* < 0.001) were associated with cancer. Levels of D-dimers (*p* = 0.001, Fig. 4c), ESR (*p* = 0.003, Fig. 4d), and CRP (*p* < 0.001, Fig. 4e) were higher in cancer patients. A trend for higher levels of LDH associated with cancer was seen (*p* = 0.052, Fig. 4f). Levels of total cholesterol, LDL, thyroid stimulatory hormone, creatinine, and non-fasting glucose were not different between groups (data not shown).

### Clinical course and outcome

**Fig. 5:**
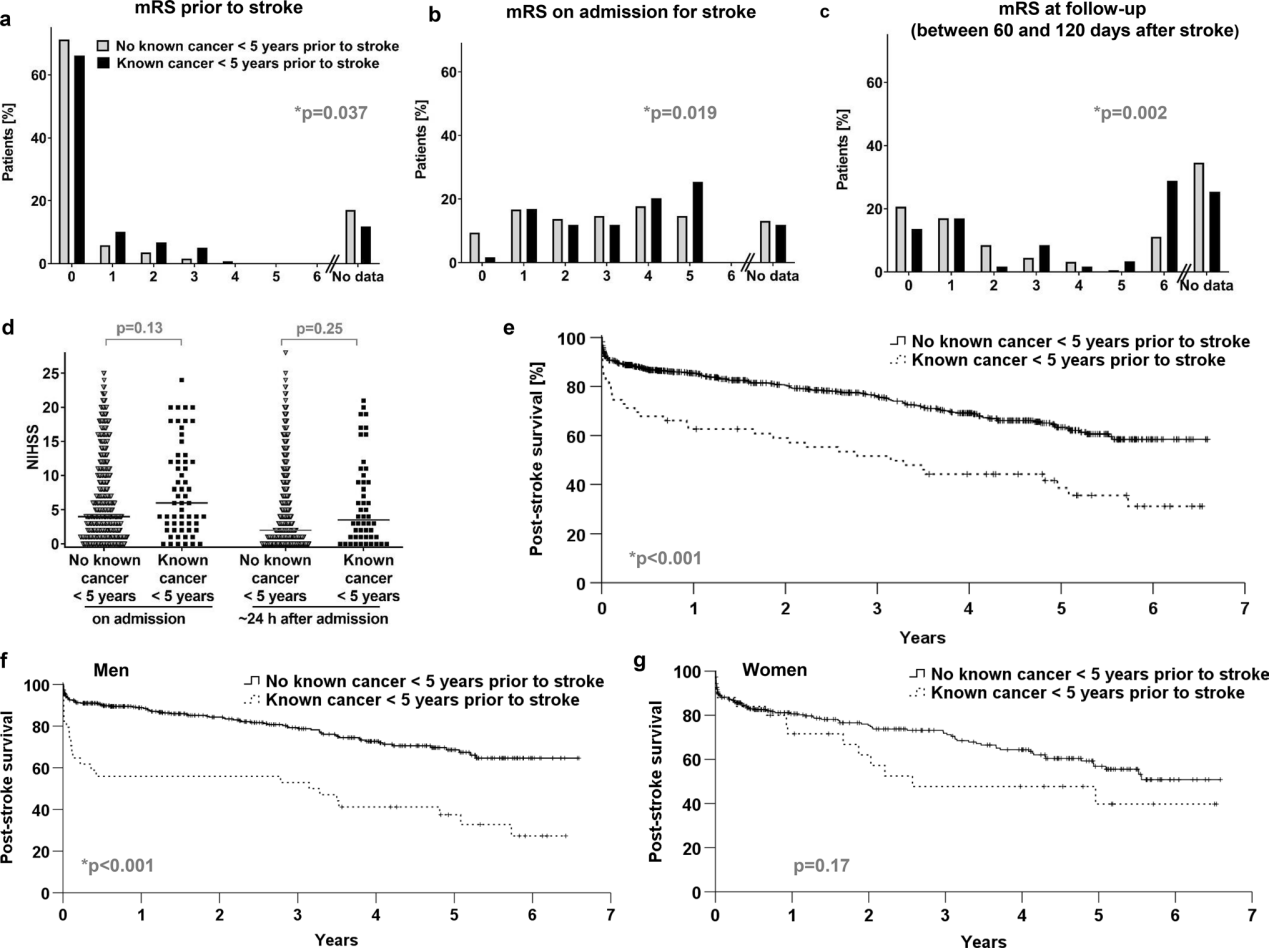
Clinical course and outcome of patients with ischemic stroke without and with known cancer. **a**–**g** Shown are modified Rankin scales (mRS) distributions (%) prior to stroke (**a**), on admission for stroke (**b**), and at follow-up after stroke (**c**) in patients without (grey bars) versus with (black bars) cancer diagnosed up to 5 years prior to stroke. Data for follow-up were included if available within 60 to 120 days after stroke or if the patient died within 120 days after stroke. Follow-up information on these patients were available by clinical visit for 71% and 50%, by indirect information via phone, relatives or documents for 20% and 23% and by documented death during hospitalization for 9% and 27% of patients without and with known cancer, respectively. NIHSS levels and their median in patients without known cancer (grey symbols, data available for n = 680 on admission, *n* = 624 after ~ 24 h) or known cancer (black symbols, data available for *n* = 59 on admission, *n* = 50 after ~ 24 h) (**d**). Kaplan–Meier survival curves of patients without (continuous line) or with (dashed line) cancer for the entire cohort (**e**) and separately for men (**f**) and women (**g**)

Next, we evaluated differences in clinical course with focus on stroke severity, degree of disability and mortality. The degree of disability as assessed by mRS was different both prior to stroke, on admission and at follow-up after stroke between patients without and with known cancer (*p* = 0.037, *p* = 0.019 and *p* = 0.002, Fig. 5a–c). When grouping patients with mRS score of ≥ 4 versus ≤ 3 on admission and at follow-up, mRS scores ≥ 4 were more frequent in cancer patients (*p* = 0.038 and *p* = 0.001). Stroke severity as evaluated by NIHSS showed no differences between both cohorts either on admission or around 24 h after stroke (Fig. 5d). In-hospital mortality was 5.7% versus 20.3% for patients without versus with cancer (*p* < 0.001). Cause of death as detailed in Table S1 was not different between groups both for in-hospital death (*p* = 0.43) and for any deaths after stroke (*p* = 0.11). Survival was worse for patients with versus without cancer (Fig. 5e, Hazard ratio (HR) 2.2, 95% CI 1.55–3.17, *p* < 0.001). Median follow-up was 2.3 years and 4.9 years for surviving patients without and with cancer. The tumor types of patients with known death are shown in Fig. 2. Medication for secondary prevention potentially affecting outcome was analyzed both at admission and at discharge without significant differences between groups as detailed in supplementary Note S1.

### Sensitivity analysis with consideration of patients with occult cancer diagnosed with cancer up to 1 year after stroke

Beyond the 11 patients with occult cancer diagnosed during in-hospital work-up of stroke that have been analyzed within the cancer group, we identified 7 patients diagnosed with cancer within the first year after stroke. For a sensitivity analysis for key parameters, we considered these 7 patients as well within the cancer group (Table S2). The results for the adapted groups were similar to the original analysis. Given the low number of patients, no separate analysis of patients with occult cancer, i.e. diagnosed after stroke, was performed.

### Activity of malignancy

We next asked whether there were differences between cancer patients with more active disease compared with cancer patients potentially cured from the disease. We compared patients diagnosed with cancer up to 5 years prior to stroke with patients diagnosed with cancer more than 5 years prior to stroke assuming that the malignancy is more active in patients with more recent diagnosis (Table S3). Patients with cancer diagnosed more than 5 years prior to stroke were older than patients with more recent diagnosis (*p* < 0.001) which may be in part interpreted by the definition of the group assignment. Previous thromboembolism was more frequent in patients with more recent cancer diagnosis (*p* = 0.023). No significant differences in stroke etiology were seen, however, there were trends for higher proportions of patients with other determined etiology and unknown etiology as well lower proportion of patients with large atherosclerosis among patients with a more recent cancer diagnosis. Regarding laboratory parameters, altered platelet counts (*p* = 0.039) and higher CRP (*p* = 0.011) were more common in patients with more recent cancer while the other analyzed parameters showed no significant differences. Degree of disability (mRS) and stroke severity (NIHSS) were comparable between both cancer groups, however, in-hospital mortality was higher in patients with more recent cancer diagnosis (*p* = 0.041).

### Analyses by sex

Since cardiovascular and cancer-associated risk profile and outcome may depend on sex, we performed separate analyses by sex. Of 25 women with stroke and cancer, the most prevalent tumors were lymphomas including hematologic diseases (28%) and breast cancer (20%). In the male cohort with cancer (34 patients), the most prevalent tumor types were prostate cancer (27%), lung cancer (15%), and lymphomas/hematologic diseases (12%). Lymph node metastases were documented in 20% and 35%, distant metastasis in 12% and 27% of women and men, respectively. Twenty-eight percent of female and 6% of male patients were on any tumor-related therapeutic regimen at the time of stroke. Out of 12 patients diagnosed with cancer during in-hospital work-up of stroke, 11 patients (92%) were male and 1 (8%) female.

Compared with the entire cohort, analysis by sex confirmed history of venous thromboembolism associated with cancer and no differences between groups regarding cardiovascular risk factors (Table S4). The results for stroke etiology and imaging characteristics in sex-specific analyses were also similar to the entire cohort. For laboratory parameters, lower hemoglobin levels (*p* < 0.001), higher levels of ESR (*p* = 0.008), and higher levels of LDH (*p* = 0.044) were associated with cancer in males but not in females. Similar to the entire cohort, reduced or elevated platelet count (*p* = 0.011 and *p* < 0.001), elevated levels of D-dimers (*p* = 0.014 and *p* = 0.027) and CRP (*p* < 0.001 and *p* = 0.011) were associated with cancer in women and in men. Regarding clinical outcome, for the entire cohort, mRS scores were different on admission and at follow-up, however, when analyzed by sex, mRS scores on admission were higher in female (*p* = 0.03) but not in male cancer patients while more unfavorable mRS at follow-up was associated with cancer in men (*p* = 0.004) but not in women. As we showed earlier, cancer was associated with poor post-stroke survival for the entire cohort (HR 2.2, 95% CI 1.55–3.18, *p* < 0.001). For females, in the entire cohort, inferior survival was observed (HR 1.4, 95% CI 1.04–1.78, *p* = 0.024). However, post-stroke survival was lower in male cancer patients compared to males without cancer (Fig. 5f, HR 3.0, 95% CI 1.88–4.76, *p* < 0.001), but not different in women with or without cancer (Fig. 5g, HR 1.5, 95% CI 0.84–2.67, *p* = 0.17). To account for these differential observations, we performed a test for interaction in bivariable logistic Cox regression analysis for risk of death with inclusion of a term of interaction (sex*known cancer < 5 years prior to stroke) which was not significant (*p* = 0.07) (Table S5).

### Multivariable analyses

We performed a model for multivariable binary logistic regression to evaluate which parameters with significant differences in the analyses above are independently associated with cancer. Since some parameters including D-dimers and erythrocyte sedimentation rate were only available for subsets of patients (70% and 67% respectively) of the cohort, we did not include these in the model. For a model including the parameters history of venous thromboembolism, hemoglobin < LLN, platelets > ULN or < LLN, and CRP ≥ ULN (data for all co-variables available for 749 patients within the study cohort of 753 patients), history of venous thromboembolism, low hemoglobin and pathological platelet count were significantly associated with known cancer (Table 3).

**Table 3:** Multivariable analyses of associations of candidate risk factors with cancer and outcome

Parameter	Odds ratio for association with cancer diagnosed up to 5 years prior to stroke	95% CI	*p* value
Binary logistic regression analysis for association of candidate parameters with known cancer: Data for all co-variables available for 749 patients			
History of venous thromboembolism: yes versus no (ref)	5.1	2.44–10.69	*< 0.001
CRP ≥ LLN^1^ mg/l versus < LLN mg/l (ref)	1.8	1.01–3.53	*0.046
Hemoglobin < LLN versus > LLN (ref)	2.5	1.39–4.47	*0.002
Platelet count < LLN or > ULN^2^ versus ≥ LLN and ≤ ULN (ref)	2.9	1.46–5.7	*0.002

Parameter	Hazard ratio for death	95% CI	*p* value
Cox logistic regression analysis for association of candidate parameters with the risk for death: Data for all co-variables available for 651 patients			
Known cancer < 5 years prior to stroke: yes versus no (ref)	1.7	1.13–2.49	*0.011
Female versus male (ref)	1.7	1.30–2.35	* < 0.001
Hemoglobin < LLN versus > LLN (ref)	2.6	1.91–3.56	* < 0.001
CRP ≥ LLN mg/l versus < LLN mg/l (ref)	1.4	1.06–1.95	*0.021
mRS on admission ≥ 4 versus < 4 (ref)	1.9	1.38–2.49	* < 0.001
Platelet count > LLN or < ULN versus ≥ LLN and ≤ ULN (ref)	1.6	1.05–2.39	*0.028
History of venous thromboembolism: yes versus no (ref)	0.9	0.54–1.63	0.82

^1^*LLN* lower level of normal

^2^*ULN* upper level of normal

Next, for the evaluation of cofactors associated with mortality after stroke, we performed a model with multivariable Cox logistic regression. We added sex due to its prognostic role in univariable analysis as shown above and mRS on admission as known prognostic factor. In the model including “known cancer < 5 years prior to stroke”, sex, mRS on admission, history of venous thromboembolism, hemoglobin, and platelet count (data for all co-variables available for 651 patients within the study cohort of 753 patients), risk for death was associated with known cancer (HR 1.7), low hemoglobin (HR 2.6), mRS on admission ≥ 4 (HR 1.9), pathologic platelet count (HR 1.6), female sex (HR 1.7), and elevated CRP (HR 1.4), while history of venous thromboembolism was not.

## Discussion

This study represents a comprehensive and exploratory analysis of patients’ demographics, history and family history, laboratory parameters, imaging findings, and clinical outcome for patients with ischemic stroke without versus with known cancer diagnosed up to 5 years prior to stroke. Differences between these groups were observed for history of venous thromboembolism, family history of cancer, and selected laboratory parameters including higher levels of D-dimers, ESR, CRP, pathologically low or high levels of platelets and lower levels of hemoglobin associated with cancer (Table 2; Fig. 4). Differences in mRS and higher mortality were linked to cancer (Fig. 5). There were sex-specific differences mainly with regard to inferior survival associated with cancer in men (Fig. 5; Table S4).

The results and interpretation of this study depend on the group assignment based on cancer diagnosis up to 5 years prior to stroke. We included patients with cancer diagnosed during the in-hospital work-up of stroke due to the temporal proximity of both diagnoses. Previous studies differ in their criteria to group cancer patients: With the term “active cancer” several authors referred to a cancer diagnosis, metastasis of known cancer, cancer recurrence, or cancer treatment, any of them present within 6 or 12 months prior to stroke [8, 10, 14, 21] with some studies additionally analyzing patients with “inactive cancer” if the criteria for “active cancer” were not fulfilled [8, 14, 15]. Since timing of treatment and diagnosis may also represent selection bias and since the risk of stroke is increased up to 10 years after cancer diagnosis [4], we selected a broader approach by including cancer patients diagnosed up to 5 years prior to stroke. The spectrum of tumor types (Fig. 2) is comparable to previous studies [8, 10, 13, 14, 21, 22], however, some excluded hematological malignancies [7, 23]. Age was not different in stroke patients without or with cancer (Table 2). Some previous studies reported higher age [9, 10], and others linked younger age to cancer in stroke patients [15]. An epidemiologic study showed an increased stroke incidence in older cancer patients, however, younger cancer patients with stroke had higher mortality [22]. We found no differences in history of cardiovascular risk factors (Table 2) also including smoking as common risk factor both for cardiovascular disease and cancer. Some studies suggested hypertension [7, 8], diabetes mellitus [8], and hyperlipidemia [7, 9, 10] to be associated with cancer in stroke patients, while others did not [11]. We observed a higher rate of previous venous thromboembolic events in tumor patients as a potential result of a cancer-associated hypercoagulability (Table 2). This was similarly shown by others, too [7, 9, 12]. There are no published data on family history of patients with stroke and cancer. We observed a higher rate of family history of cancer in stroke patients with cancer while other parameters of family history were not different between groups (Table 2), however, results are limited by the retrospective design and data availability only in subsets of patients.

Stroke etiology as classified by TOAST was not different between groups (Fig. 3a) while some studies reported an association of the cryptogenic subtype with cancer [7, 8, 15], however others found cardioembolic strokes more frequent in cancer patients [24]. Previous studies linked stroke in multiple vessel territories to cancer [7, 8, 15]. However, we did not identify differences in the incidence of ischemic lesions in ≥ 2 versus < 2 vessel territories between groups (Fig. 3b). So far, little is known about the incidence of large vessel occlusions in stroke patients with cancer. Regarding a potential tumor-associated hypercoagulability, tumor patients might be at higher risk for large vessel occlusions. However, we did not confirm this hypothesis (Fig. 3c).

Regarding laboratory values (Fig. 4), lower levels of hemoglobin [10], higher levels of D-dimers [7, 8, 10, 21], elevated ESR [14], and elevated levels of CRP [8, 10, 14, 15] have been linked to cancer patients. In our cohort, both very high and low platelets were associated with cancer (Fig. 4b) which was not found by others [9, 14]. However, in the context of unprovoked venous thromboembolism, elevated platelet counts were associated with occult cancer [25]. There are only scarce longitudinal data on clinical indexes including mRS and NIHSS as outcome measures in tumor patients with stroke. One case control study of 69 stroke patients found no difference in mRS ≤ 3 versus ≥ 4 on admission and at discharge, but reported higher in-hospital mortality associated with cancer [18]. Another study also observed higher in-hospital mortality for patients with stroke and cancer with covariables of higher NIHSS and higher CRP at admission [15]. Other authors reported no differences in short-term prognosis including neurological improvement, mRS 0–2 at discharge, and in-hospital mortality [14]. In our cohort, mRS scores ≥ 4 were more frequent in cancer patients both at admission and at follow-up while NIHSS was not different between groups. Higher in-hospital mortality but also poorer survival after ischemic stroke were linked to cancer (Fig. 5).

Known cancer up to 5 years prior to stroke represents an independent prognostic cofactor for mortality after stroke with additional prognostic cofactors including low hemoglobin, elevated CRP, pathologic platelet count, female sex and mRS on admission ≥ 4 (Table 3). The association of lower hemoglobin as a cofactor linked to higher mortality reflects previous observations that anemia is linked to poor prognosis in stroke [26, 27]. In line with our data, several authors found inferior post-stroke survival associated with female sex [28–32] while others did not [33] or reported higher short-term survival for women after multivariable adjustments [34]. In our study, in sex separate-analyses, we observed that inferior post-stroke survival was associated with cancer in men but not significantly different in women with versus without cancer (Fig. 5). We acknowledge that with regard to the inferior survival of women in the entire cohort and non-significant testing for interaction of sex and cancer in this context (Table S5), this observation has to be interpreted with caution and requires validation in a larger cohort. However, the poor outcome of male tumor patients may, at least partly, be interpreted by lower rates of metastatic disease in women. However, a higher proportion of women (28%) than men (6%) were on a tumor-related therapeutic regimen at diagnosis of stroke. Interestingly, the higher rate of men diagnosed with cancer during in-hospital work-up of stroke may reflect a lack of early cancer diagnosis. Epidemiologic data of cancer patients in the US with 1% suffering from lethal stroke showed no sex-specific differences regarding the risk to die of stroke [22]. Beyond this, no data on sex-associated characteristics in cancer patients with stroke are available, however, this may be relevant since both diseases exhibit sex-associated differences in respective risk profiles.

The strengths of our study represent the comprehensive analysis of numerous parameters for a cancer-related association in stroke patients and the incorporation of relevant parameters for analysis of post-stroke survival. Limitations include the retrospective study design with heterogeneity of patients, availability of some readouts only in subsets of patients, and especially in subgroups rather low patient numbers.

## Conclusions

This study extends the knowledge on characteristics and outcome of cancer patients with ischemic stroke. Particularly, history of venous thromboembolism, low hemoglobin, altered platelet count and elevated CRP were associated with cancer in stroke patients, as well as higher in-hospital mortality and inferior post-stroke survival. Considering cancer as a cofactor for post-stroke outcome may impact clinical decision making and risk stratification.

## Supplementary Information

Below is the link to the electronic supplementary material.

Supplementary file1 (DOCX 21 KB)

Supplementary file2 (DOCX 17 KB)

Supplementary file3 (DOCX 39 KB)

Supplementary file4 (DOCX 44 KB)

Supplementary file5 (DOCX 21 KB)

Supplementary file6 (DOCX 18 KB)

## Data Availability

Anonymized data not published within the article will be shared on reasonable request from any qualified investigator provided that it is in line with the requirements of the institutional review board approval.

## Declarations

### Code availability

No codes were used for this work.
