# Supplementary material for: Cancer is associated with inferior outcome in patients with ischemic stroke
Source: J Neurol. 2021 May 4;268(11):4190–202. doi: 10.1007/s00415-021-10528-3 (PMC8505392; doi:10.1007/s00415-021-10528-3)
Supplement: Supplementary file 1 — Supplementary file1 (DOCX 21 KB) [file 415_2021_10528_MOESM1_ESM.docx]

**Table S1: Cause of death for patients without known and known cancer up to 5 years prior to stroke**

|  | **No known cancer**  **< 5 years prior to stroke** | **Known cancer**  **< 5 years prior to stroke** |
| --- | --- | --- |
| **Deaths**  **Cause of death**   - Cardiovascular - Cancer - Other - Unknown | 177/694 patients (25.5%)  41.2%  0.6%  9.0%  49.2% | 36/59 patients (61%)  36.1%  5.6%  5.6%  52.8% |
| **Deaths during hospitalisation**  **Cause of death**   - Cardiovascular - Cancer - Other - Unknown | 40/694 patients (5.8%)  95.0%  0%  5.0%  0% | 12/59 patients (20.3%)  100%  0%  0%  0% |
