# Supplementary material for: Cancer is associated with inferior outcome in patients with ischemic stroke
Source: J Neurol. 2021 May 4;268(11):4190–202. doi: 10.1007/s00415-021-10528-3 (PMC8505392; doi:10.1007/s00415-021-10528-3)
Supplement: Supplementary file 2 — Supplementary file2 (DOCX 17 KB) [file 415_2021_10528_MOESM2_ESM.docx]

**Table S2: Characteristics of patients with known cancer up to 5 years prior to stroke or up to 1 year after stroke compared with patients with no known cancer in this period: A sensitivity analysis**

|  | **No known cancer within 5 years prior to stroke and up to 1 year after stroke:**  **687 patients** | **Known cancer within 5 years prior to stroke and up to 1 year after stroke:**  **66 patients** | **p value** |
| --- | --- | --- | --- |
| **Sex: n (%)**   - Male - Female | 391 (56.9%)  296 (43.1%) | 38 (57.6%)  28 (42.4%) | 0.92 |
| **Age:** Median (Min-max) | 73 (21-100) | 74 (27-94) | 0.07 |
| **History of venous thromboembolism: n (%)** | 34 (4.9%) | 14 (21.2%) | *<0.001 |
| **TOAST classification**   - Large artery atherosclerosis - Cardiac embolism - Small vessel disease - Other determined etiology - Unknown or more than 1 possible etiology | 114 (16.6%)  260 (37.8%)  68 (9.9%)  43 (6.3%)  202 (29.4%) | 5 (7.6%)  28 (42.4%) 8 (12.1%)  6 (9.1%)  19 (28.8%) | 0.35 |
| **Vessel territories with acute ischemic lesions: n (%)**   - < 2 - ≥ 2 | 559 (81.4%)  128 (18.6%) | 51 (77.3%)  15 (22.7%) | 0.42 |
| **Large vessel occlusion**   - Yes - No - No data | 236 (34.4%)  443 (64.5%)  8 (1.2%) | 25 (37.9%)  40 (60.6%)  1 (1.5%) | 0.55 |
| **Laboratory parameters^1^** |  |  |  |
| - Hemoglobin: Median (Min-max) | 139 (58-186) | 132 (61-194) | *0.001 |
| - Platelet count: n (%)   < LLN^2^  ≥ LLN and ≤ ULN^3^  > ULN | 36 (5.2%)  632 (92%)  19 (2.8%) | 7 (10.6%)  49 (74.2%)  10 (15.2%) | *<0.001 |
| - White blood count: n (%)   < ULN  ≥ ULN | 502 (73.1%)  185 (26.9%) | 45 (28.2%)  21 (31.8%) | 0.40 |
| - D dimer: Median (Min-max)   - Data available for: n (%) | 0.83 (0.08-20)  482 (70.2%) | 1.4 (0.38-20)  43 (65.2%) | *0.001 |
| - Erythrocyte sedimentation rate:   Median (Min-max)   - - Data available for: n (%) | 10 (1-130)  457 (66.5%) | 16 (1-80)  46 (69.7%) | *0.012 |
| - C-reactive protein: Median (Min-max)   - Data available for: n (%) | 2.4 (0-358)  683 (99.4%) | 4.4 (0.3-374)  66 (100%) | *<0.001 |
| - Lactate Dehydrogenase: Median (Min-max)   - Data available for: n (%) | 368 (95-2245)  575 (83.7%) | 386 (156-1606)  59 (89.4%) | 0.081 |
| **Modified Rankin Scale**   - Prior to stroke   - 0   - 1   - 2   - 3   - 4   - 5   - Data available for: n (%) - On admission for stroke   - 0   - 1   - 2   - 3   - 4   - 5   - Data available for: n (%) - At follow-up (between 60 and 120 days after stroke)   - 0   - 1   - 2   - 3   - 4   - 5   - 6   - Data available for: n (%) | 490 (71.3%)  41 (6.0%)  24 (3.5%)  11 (1.6%)  5 (0.7%)  0 (0%)  571 (83.1%)  65 (9.5%)  115 (16.7%)  94 (13.7%)  99 (14.4%)  123 (17.9%)  101 (14.7)  597 (86.9%)  140 (20.4%)  118 (17.2%)  58 (8.4%)  31 (4.5%)  21 (3.1%)  4 (0.6%)  77 (11.1%)  449 (65.4%) | 43 (65.2%)  6 (9.1%)  5 (7.6%)  3 (4.5%)  0 (0%)  0 (0%)  57 (86.4%)  1 (1.5%)  11 (16.7%)  8 (12.1%)  10 (15.2%)  12 (18.2%)  16 (24.2%)  58 (87.9%)  11 (16.7%)  10 (15.2%)  2 (3%)  5 (7.6%)  2 (3.0%)  2 (3.0%)  17 (25.8%)  49 (74.2%) | *0.034  *0.023  *0.009 |
| - **NIHSS on admission:** Median (Min-max)   - Data available for: n (%) | 4 (0-25)  673 (98.0%) | 6 (0-24)  66 (100%) | 0.17 |
| - **NIHSS ~ 24h after admission:** Median (Min-max)   - Data available for: n (%) | 2 (0-28)  617 (89.8%) | 3 (0-21)  57 (86.4%) | 0.47 |

^1^ If not otherwise indicated, data for the entire cohort were available

^2^ LLN: lower level of normal

^3^ ULN: upper level of normal
