# Supplementary material for: Cancer is associated with inferior outcome in patients with ischemic stroke
Source: J Neurol. 2021 May 4;268(11):4190–202. doi: 10.1007/s00415-021-10528-3 (PMC8505392; doi:10.1007/s00415-021-10528-3)
Supplement: Supplementary file 3 — Supplementary file3 (DOCX 39 KB) [file 415_2021_10528_MOESM3_ESM.docx]

**Table S3: Comparison of characteristics of cancer patients with diagnosis within 5 years prior to stroke versus more than 5 years prior to stroke**

|  | **Known cancer more than 5 years prior to stroke:**  **74 patients** | **Known cancer within 5 years prior to stroke: 59 patients** | **p value** |
| --- | --- | --- | --- |
| **Sex: n (%)**   - Male - Female | 38 (51.4%)  36 (48.6%) | 34 (57.6%)  25 (42.4%) | 0.47 |
| **Age:** Median (Min-max) | 82 (50-95) | 73 (27-91) | *<0.001 |
| **History of venous thromboembolism: n (%)** | 6 (8.1%) | 13 (22.0%) | *0.023 |
| **TOAST classification**   - Large artery atherosclerosis - Cardiac embolism - Small vessel disease - Other determined etiology - Unknown or more than 1 possible etiology | 13 (17.6%)  37 (50%)  6 (8.1%)  1 (1.4%) 17 (23%) | 4 (6.8%)  26 (44.1%)  5 (8.5%) 6 (10.2%)  18 (30.5%) | 0.07 |
| **Vessel territories with acute ischemic lesions: n (%)**   - < 2 - ≥ 2 | 59 (79.7%)  15 (20.3%) | 45 (76.3%) 14 (23.7%) | 0.63 |
| **Large vessel occlusion**   - Yes - No - No data | 26 (35.1%)  43 (58.1%)  5 (6.8%) | 23 (39.0%)  35 (59.3%)  1 (1.7%) | 0.82 |
| **Laboratory parameters^1^** |  |  |  |
| - Hemoglobin:   - Median (Min-max) | 133 (58-169) | 132 (61-194) | 0.69 |
| - Platelet count: n (%)   < LLN^2^  ≥ LLN and ≤ ULN^3^  > ULN | 6 (8.1%)  66 (89.2%)  2 (2.7%) | 7 (11.9%)  44 (74.6%)  8 (13.6%) | *0.039 |
| - White blood count: n (%)   < ULN  ≥ ULN | 57 (77%)  17 (23%) | 41 (69.5%) 18 (30.5%) | 0.33 |
| - D dimer: Median (Min-max)   - Data available for: n (%) | 1.20 (0.19-20)  50 (67.6%) | 1.42 (0.38-20)  38 (64.4%) | 0.24 |
| - Erythrocyte sedimentation rate:   Median (Min-max)   - - Data available for: n (%) | 18 (2-85)  47 (63.5%) | 16 (2-80)  40 (67.8%) | 0.55 |
| - C-reactive protein: Median (Min-max) | 2.7 (0.3-144) | 4.5 (0.3-374) | *0.011 |
| - Lactate Dehydrogenase: Median (Min-max)   - Data available for: n (%) | 384 (193-995)  56 (75.7%) | 386 (156-1606)  53 (89.8%) | 0.28 |
| **Modified Rankin Scale**   - Prior to stroke   - 0   - 1   - 2   - 3   - 4   - 5   - Data available for: n (%) - On admission for stroke   - 0   - 1   - 2   - 3   - 4   - 5   - Data available for: n (%) - At follow-up (between 60 and 120 days after stroke)   - 0   - 1   - 2   - 3   - 4   - 5   - 6   - Data available for: n (%) | 47 (63.5%)  11 (14.9%)  1 (1.4%)  3 (4.1%)  1 (1.4)  0 (0%) 63 (85.1%)  1 (1.4%)  19 (25.7%)  7 (9.5%)  7 (9.5%)  15 (20.3%)  19 (25.7%)  68 (91.9%)  10 (13.5%)  11 (14.9%)  2 (2.7%)  2 (2.7%)  3 (4.1%)  0 (0%)  22 (29.7%)  50 (67.6%) | 39 (66.1%)  6 (10.2%)  4 (6.8%)  3 (5.1%)  0 (0%)  0 (0%) 52 (88.1%)  1 (1.7%)  10 (16.9%)  7 (11.9%)  7 (11.9%)  12 (20.3%)  15 j(25.4%)  52 (88.1%)  8 (13.6%)  10 (16.9%)  1 (1.7%)  5 (8.5%)  1 (1.7%)  2 (3.4%)  17 (28.8%)  44 (74.6%) | 0.95  0.62  0.89 |
| - **NIHSS on admission:** Median (Min-max)   - Data available for: n (%) | 6 (0-25)  74 (100%) | 6 (0-24)  59 (100%) | 0.71 |
| - **NIHSS ~ 24h after admission:** Median (Min-max)   - Data available for: n (%) | 4 (0-22)  68 (91.9%) | 4 (0-21)  50 (84.7%) | 0.58 |
| **In-hospital mortality** | 6 (8.1%) | 12 (20.3%) | *0.041 |

^1^ If not otherwise indicated, data for the entire cohort were available

^2^ LLN: lower level of normal

^3^ ULN: upper level of normal
