# Supplementary material for: Cancer is associated with inferior outcome in patients with ischemic stroke
Source: J Neurol. 2021 May 4;268(11):4190–202. doi: 10.1007/s00415-021-10528-3 (PMC8505392; doi:10.1007/s00415-021-10528-3)
Supplement: Supplementary file 4 — Supplementary file4 (DOCX 44 KB) [file 415_2021_10528_MOESM4_ESM.docx]

**Table S4: Subgroup analysis stratified by sex**

|  | **Female** | | | **Male** | | |
| --- | --- | --- | --- | --- | --- | --- |
|  | **No known cancer <5 years prior to stroke: n=299^1^** | **Known cancer <5 years prior to stroke: n=25^1^** | **p value** | **No known cancer <5 years prior to stroke: n=395^1^** | **Known cancer <5 years prior to stroke: n=34^1^** | **p value** |
| **Age:** Median (Range) | 78 (27-96) | 79 (54-91) | 1.0 | 69 (21-100) | 72 (27-89) | 0.08 |
| **Body mass index**: Median (Range)  data available for (n) | 24.8 (15.1-54.1)  286 | 26.1 (16.7-34.6)  24 | 0.10 | 26.0 (15.3-41.5)  383 | 25.3 (19.0-30.9)  32 | 0.21 |
| **History** |  |  |  |  |  |  |
| Arterial hypertension, n (%) | 215 (71.9%) | 18 (72.0%) | 0.99 | 253 (64.1%) | 26 (76.5%) | 0.15 |
| Diabetes mellitus, n (%) | 41 (13.7%) | 4 (16.0%) | 0.75 | 62 (15.7%) | 9 (26.5%) | 0.11 |
| Hyperlipidemia, n (%) | 150 (49.8%) | 9 (36.0%) | 0.17 | 188 (47.6%) | 17 (50.0%) | 0.79 |
| Active or previous smoking, n (%)  data available for (n) | 89 (33.7%)  264 | 4 (18.2%)  22 | 0.38 | 209 (57.3%)  365 | 21 (70.0%)  30 | 0.17 |
| Atrial fibrillation, n (%) | 72 (24.1%) | 9 (36.0%) | 0.19 | 68 (17.2%) | 7 (20.6%) | 0.62 |
| Heart disease (not otherwise specified), n (%) | 84 (28.1%) | 10 (40.0%) | 0.21 | 123 (31.1%) | 13 (38.2%) | 0.39 |
| Ischemic stroke, n (%) | 38 (12.7%) | 4 (16.0%) | 0.64 | 62 (15.7%) | 6 (17.6%) | 0.77 |
| Intracranial hemorrhage, n (%) | 8 (2.7%) | 1 (4.0%) | 0.70 | 10 (2.5%) | 2 (5.9%) | 0.26 |
| Transient ischemic attack, n (%) | 20 (6.7%) | 0 (0%) | 0.18 | 21 (5.3%) | 0 (0%) | 0.17 |
| Venous thromboembolism, n (%) | 19 (6.4%) | 7 (28.0%) | *<0.001 | 16 (4.1%) | 6 (17.6%) | *0.001 |
| **TOAST classification,** n (%)  (1) Large artery atherosclerosis  (2) Cardiac embolism  (3) Small vessel disease  (4) Other determined etiology  (5) Unknown etiology or more than 1 possible etiology | 38 (12.7%)  125 (41.8%)  32 (10.7%)  14 (4.7%)  90 (30.1%) | 1 (4.0%)  15 (60.0%)  0 (0%)  3 (12.0%)  6 (24.0%) | 0.07 | 77 (19.5%)  137 (34.7%)  39 (9.9%)  29 (7.3%)  113 (28.6%) | 3 (8.8%)  11 (32.4%)  5 (14.7%)  3 (8.8%)  12 (35.3%) | 0.51 |
| **Imaging characteristics** |  |  |  |  |  |  |
| Ischemic lesions in  < 2 vessel territories, n (%)  ≥ 2 vessel territories, n (%) | 240 (80.3%)  59 (19.7%) | 20 (80.0%)  5 (20.0%) | 0.97 | 325 (82.3%)  70 (17.7%) | 25 (73.5%)  9 (26.5%) | 0.21 |
| Large vessel occlusion, n (%)  data available for (n) | 112 (37.7%)  297 | 11 (44.0%)  25 | 0.53 | 126 (32.4%)  391 | 12 (36.4%)  33 | 0.64 |
| **Laboratory parameters** |  |  |  |  |  |  |
| Hemoglobin (g/l): Median (Min-max) | 132 (77-171) | 137 (61-150) | 0.60 | 144 (58-186) | 128 (95-194) | *<0.001 |
| Platelet count  <LLN^2^, n (%)  ≥LLN/μl and ≤ ULN^3^, n (%)  >ULN/μ, n (%) | 185 (4.3%)  276 (92.3%)  10 (3.3%) | 1 (4.0%)  20 (80.0%)  4 (16.0%) | *0.011 | 24 (6.1%)  361 (91.4%)  10 (2.5%) | 6 (17.6%)  24 (70.6%)  4 (11.8%) | *<0.001 |
| White blood count  ≤ULN, n (%)  >ULN/μl, n (%) | 224 (71.6%)  85 (28.4%) | 18 (72.0%)  7 (28.0%) | 0.96 | 292 (73.9%)  103 (26.1%) | 23 (67.6%)  11 (32.4%) | 0.43 |
| D-dimers (mg/l): Median (Min-max)  data available for (n) | 1.2 (0.09-20)  204 | 1.8 (0.7-9.8)  18 | *0.014 | 0.7 (0.08-13.4)  283 | 0.9 (0.4-20)  20 | *0.027 |
| Erythrocyte sedimentation rate (mm/h): Median (Min-max)  data available for (n) | 14 (2-100)  187 | 19 (5-70)  16 | 0.16 | 9 (1-130)  276 | 12 (2-85)  24 | *0.008 |
| C reactive protein (mg/l): Median (Min-max)  data available for (n) | 2.7 (0-277)  297 | 4.5 (1.5-198)  25 | *0.001 | 2.3 (0-358)  393 | 4.4 (0.3-374)  31 | *0.011 |
| Lactate dehydrogenase: Median (Min-max)  data available for (n) | 382 (177-2245)  250 | 403 (190-752)  22 | 0.57 | 359 (95-1122)  324 | 383 (156-1606)  30 | *0.044 |
| **Clinical course and outcome** |  |  |  |  |  |  |
| mRS prior to stroke: Median (Min-max)  data available for (n) | 0 (0-4)  248 | 0 (0-3)  21 | 0.43 | 0 (0-4)  328 | 0 (0-3)  31 | *0.023 |
| mRS on admission for stroke:  Median (Min-max)  data available for (n) | 3 (0-5)  258 | 4 (0-5)  22 | *0.032 | 3 (0-5)  345 | 3 (1-5)  30 | 0.21 |
| mRS at follow-up: Median (Min-max)  data available for (n) | 1 (0-6)  196 | 3 (0-6)  17 | 0.20 | 1 (0-6)  258 | 5 (0-6)  27 | *0.004 |
| NIHSS on admission (Min-max)  data available for (n) | 5 (0-25)  296 | 6 (0-20)  25 | 0.69 | 4 (0-25)  384 | 7 (0-24)  34 | 0.10 |
| NIHSS about 24h after admission  Median (Min-max)  data available for (n) | 3 (0-25)  268 | 3 (0-20)  31 | 0.79 | 2 (0-28)  356 | 4 (0-21)  28 | 0.19 |

^1^If data are available only for subsets of patients, the numbers of patients for whom data are available are indicated and percentages refer to the patients with available data and not to the entire cohort. If no separate “n” is indicated, percentages refer to the total number of female or male patients with no known or known cancer, respectively.

^2, 3^  LLN: lower level of normal, ULN: upper level of normal
