# Supplementary material for: Cancer is associated with inferior outcome in patients with ischemic stroke
Source: J Neurol. 2021 May 4;268(11):4190–202. doi: 10.1007/s00415-021-10528-3 (PMC8505392; doi:10.1007/s00415-021-10528-3)
Supplement: Supplementary file 5 — Supplementary file5 (DOCX 21 KB) [file 415_2021_10528_MOESM5_ESM.docx]

**Table S5: Bivariable Cox logistic regression analysis for cancer and sex**

| **Parameter** | **Hazard Ratio for death** | **95% CI** | **p value** |
| --- | --- | --- | --- |
| Known cancer < 5 years prior to stroke: yes versus no (ref) | 2.2 | 1.55-3.17 | *<0.001 |
| Sex: Female versus male (ref) | 1.4 | 1.04-1.78 | *0.027 |
| **Test for interaction** |  |  |  |
| “Sex” * “Known cancer < 5 years prior to stroke” | 0.5 | 0.24-1.05 | 0.07 |
| Known cancer < 5 years prior to stroke: yes versus no (ref) | 3.01 | 1.90-4.79 | *<0.001 |
| Sex: Female versus male (ref) | 1.5 | 1.13-2.04 | *0.006 |
