# Supplementary material for: Cancer is associated with inferior outcome in patients with ischemic stroke
Source: J Neurol. 2021 May 4;268(11):4190–202. doi: 10.1007/s00415-021-10528-3 (PMC8505392; doi:10.1007/s00415-021-10528-3)
Supplement: Supplementary file 6 — Supplementary file6 (DOCX 18 KB) [file 415_2021_10528_MOESM6_ESM.docx]

**Supplementary Note S1:**

Medication for secondary prevention of stroke on admission was not different comparing patients receiving either no medication, single antiplatelet therapy, dual antiplatelet therapy, therapeutic anticoagulation, therapeutic anticoagulation plus single antiplatelet therapy or therapeutic anticoagulation plus dual antiplatelet therapy (p=0.51). Similarly, comparison of patients without any medical secondary prevention versus all others (p=0.72), single antiplatelet therapy versus all others (p=0.55), and any therapeutic anticoagulation versus all others (p=0.13) showed no differences between groups. Medication for secondary prevention was analysed also at discharge excluding those patients with in-hospital death, i.e. evaluating 654 without and 47 patients with cancer. Comparison of patients receiving either no medical secondary prevention, single antiplatelet therapy, dual antiplatelet therapy, therapeutic anticoagulation, therapeutic anticoagulation plus single antiplatelet therapy or therapeutic anticoagulation plus dual antiplatelet therapy showed no significant differences (p=0.63). Similarly, comparison of patients receiving single antiplatelet therapy versus all others (p=0.08) and receiving any therapeutic anticoagulation versus all others showed no difference (p=0.96).
